# Outcome of Colonic Surgery in Elderly Patients with Colon Cancer

**DOI:** 10.1155/2010/865908

**Published:** 2010-06-13

**Authors:** E. Hermans, P. M. van Schaik, H. A. Prins, M. F. Ernst, P. J. L. Dautzenberg, K. Bosscha

**Affiliations:** ^1^Department of Surgery, Jeroen Bosch Hospital, Nieuwstraat 54, 5211's-Hertogenbosch, The Netherlands; ^2^Department of Geriatrics, Jeroen Bosch Hospital, Nieuwstraat 54, 5211's-Hertogenbosch, The Netherlands

## Abstract

*Introduction*. Colonic cancer is one of the most commonly diagnosed malignancies and most often occurs in patients aged 65 years or older. *Aim*. To evaluate the outcome of colonic surgery in the elderly in our hospital and to compare five-year survival rates between the younger and elderly patients. *Methods*. 207 consecutive patients underwent surgery for colon cancer. Patients were separated in patients younger than 75 and older than 75 years. *Results*. Elderly patients presented significantly more (*P* < .05) as a surgical emergency, had a longer duration of admission and were more often admitted to the ICU (*P* < .01). Also, elderly patients had significant more co-morbidities, especially cardiovascular pathology (*P* < .01). Post-operative complications were seen more often in the elderly, although no significant difference was seen in anastomotic leakage. The five-year survival rate in the younger group was 62% compared with 36% in the elderly (*P* < .05). DFS was 61% in the younger patients compared with 32% in the elderly (*P* < .05). *Conclusion*. Curative resection of colonic carcinoma in the elderly is well tolerated and age alone should not be an indication for less aggressive therapy. However, the type and number of co-morbidities influence post-operative mortality and morbidity.

## 1. Introduction

Colonic cancer is one of the most commonly diagnosed malignancies in men and women in developed countries. The disease rarely occurs before age of 40 and the risk of colonic cancer is the highest around age of 70. Seventy-five percent of colon tumors are found in patients aged 65 years or older [1].

The incidence of colonic cancer has increased in the last decades. With the increase of age in the general population in developed countries the next future decades, the number of elderly patients who present with this disease will increase [2]. Unfortunately, most elderly patients who develop colonic cancer also have significant other comorbidities such as cardiovascular and pulmonary diseases, which increase the operative risk and the risk of postoperative morbidity and mortality [3]. Other factors that contribute to poor outcome of surgery in the elderly are delayed presentation and more advanced disease [4].

Therefore, curative surgery of colonic cancer in elderly patients is debatable, especially in the very elderly patients, who have limited prospects of survival. Some authors promote extensive surgery, including multistage procedures, as performed in younger patients [5, 6]; others promote less aggressive surgery [7, 8].

The aim of this study was to evaluate the outcome of colonic surgery in the elderly in our hospital to determine the best treatment strategy in this patient category.

## 2. Patients and Methods

In the period January 1999–January 2004, 207 consecutive patients underwent surgery for stages I–III colonic cancer. Patients with rectal cancer and patients that presented with distant metastases were excluded. All patients were separated into two groups, one group with patients younger than 75 years and one group with patients older than 75 years.

This separation was based on several other publications concerning this subject [2, 9–11].

The following data were collected for each patient: gender, age, location, and characteristics of the tumour, type of operation, duration of admittance, ICU admittance, comorbidity, complications, type of ileostomy/colostomy, disease-free survival, and overall survival.

Comorbidity was classified according to an adapted version of Charlson et al. and was divided into previous malignancies, COPD, cardiovascular disease, cerebrovascular disease, hypertension, diabetes, and other [12]. 

Followup was conducted according to the criteria of the Dutch Cancer Centre recommendations.

Statistical analyses were performed using SPSS 13.0 (SPSS, Chicago, Ill, USA). Mann-Whitney *U* test was performed to establish significance between the number of days of admittance in both groups. Chi-square test was performed to determine significance in nonparametric variables. Kaplan-Meier curves were used in the disease-free survival and overall survival. *P* < .05 was found to be significant.

## 3. Results

Seventy-four of 207 patients (36%) were 75 years or older when operated upon, male: female 30 : 44. The mean age of the elderly patients was 80 years [range 75–100] compared with a mean age of 62 years in the group < 75 years [range 38–74]. Sixteen of 74 (22%) patients presented as a surgical emergency and were operated upon within 24 hours after presentation.

In the younger group, 11 patients (9%) presented as a surgical emergency (*P* < .05).

In the elderly group thirteen patients (81%) had abdominal complaints for less than 3 weeks.

In the other 3 patients there was serious patient delay because all had significant complaints for more than 6 weeks and refused to seek medical care. In the younger patients that were operated upon in an emergency setting, none of them had symptoms a month prior to operation.

No differences were found in tumor location, depth of invasion, or Dukes' stage.

Patients' characteristics and tumor features are summarized in Table 1.

The mean number of days of admittance to the hospital in the elderly group was 16.5 days [range 2–68]. The mean number of days of admittance to the ICU was 2.3 days [range 0–38]. In the group <75 years the mean number of days of admittance was 14.9 days [4–84], and the mean number of days of admittance to the ICU was 1.3 [0–30] (*P* < .01). Forty percent of the elderly patients had to be admitted to the ICU compared with 18% in the younger group (*P* < .01).

Elderly patients more often presented with significant comorbidity (Table 2) especially more cardiovascular pathology (*P* < .05) and demential syndrome (*P* < .01). Also, elderly patients presented significantly more often with more than one type of comorbidity according to the Charlson classification (*P* < .05) when compared with the younger patients.

Table 3 shows the performed types of surgery. These did not significantly differ between the elderly and the younger patients. In total 5 subtotal colectomies were performed, one in an elderly patient, who had a large tumour in the sigmoid and a blow-out of the cecum. The other 4 subtotal colectomies were performed in young patients with Lynch's Syndrome (HNPCC).

In 85% of the elderly patients and in 86% of the younger patients, a primary anastomosis was made. No significant difference was found in the number and types of ileo- and colostomies.

Complications were seen in 32 younger patients (24%) and in 37 elderly patients (50%) (*P* < .01). No difference was found in major complications as anastomotic leakage, fascia dehiscence, or intraabdominal abscesses.

Significant however was the higher number of pneumonias, wound infections, and minor complications (i.e., urinary tract infection, and electrolyte disturbances) in the elderly group (*P* < .05). Also, the number of deliriums was significantly higher in the elderly group (*P* < .01). Complications are listed in Table 4.

The in-hospital mortality was 16% (12 patients) in the elderly and 5% (6 patients) in the younger group (*P* < .01). The mean age of the patients that died in the elderly group was 84 years and 67 years in the younger group. 

**Figure 1:**
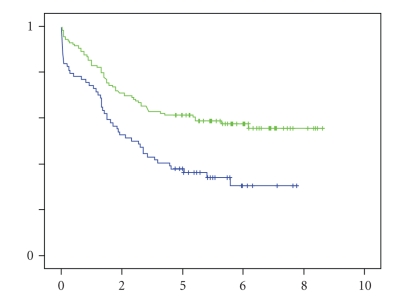
Kaplan-Meier 5-year overall-survival curve. Blue solid line for patients > 75 years and green solid line for patients < 75 years.

Of all elderly patients that died, 5 died of sepsis and multiple organ failure, 3 due to pneumonia or respiratory failure, 2 due to myocardial infarction, and 1 due to a high gastrointestinal bleeding. In the younger patients, 3 died of sepsis and 2 due to respiratory failure. A palliative resection was performed in 2 patients, each in one group. Unfortunately, these 2 patients were in such worse condition that they died after prolonged ICU admittance. The five-year survival rate of patients, who were treated with curative resection, was 62% in the younger group compared with 36% in the elderly (*P* < .05) (Figure 1). When corrected for cancer-related survival, the 5-year survival rate was 63% versus 42% (NS). 

Significant however was the number of deaths during 5-year followup which were not (colonic) cancer related, in the younger group 4/51 versus 12/36 in the elderly (*P* < .01).

Only 6 patients in the elderly group received adjuvant chemotherapy (8%), while 33 patients were qualified for adjuvant therapy. In the younger group 40 patients (30%) received adjuvant therapy, while 68 patients were qualified. Besides age, the main reason not to give adjuvant treatment was a low Karnovsky index.

DFS was 61% in the younger patients compared with 32% in the elderly (*P* < .05).

## 4. Discussion

Surgical resection remains the core in curative treatment for colonic cancer. Due to the steadily expansion of the elder population in the industrialized world, surgeons will be confronted with more and more elderly patients.

In a systematic review, which was published in the Lancet in 2000, it is demonstrated that in elderly patients less often a resection of the tumour is performed than in younger patients [9].

However, studies have shown that age alone is not a significant prognostic factor in survival after colonic surgery [13, 14].

Therefore, it is better to speak of biological age rather than chronological age when assessing risk factors for surgery, which focuses more on the overall condition of the patient.

Because treatment options in colonic cancer increase, recent literature shows a decrease in resection rate between elderly and younger patients [15].

Specific problems related with elderly patients are, as mentioned earlier, a significant higher number of comorbidities, prolonged in-hospital stay, and a delayed or emergency presentation. In our study 77% of the elderly had at time of surgery one or more types of comorbidity. This corresponds with other studies, which report an incidence of 70%–85% [16, 17].

Most patients in the elderly group were classified as ASA III.

It is well documented that emergency surgery is related with a higher mortality rate than elective surgery [18, 19]. 

When we compare 5-year survival between the patients that were operated in an emergency setting with the patients who were operated electively, there was a significant difference in 5-year survival (39% versus 56%) (*P* < .01).

In-hospital stay did not significantly differ between the two groups. This is remarkable because of the high number of postoperative complications (pneumonias, cardiovascular problems, and deliriums) in the elderly group. Delirium is a known, serious complication with high incidence in elderly patients. It increases the chance of other complications, a longer duration of admittance, worse recovery (physically and mentally), and a higher mortality [20, 21].

In our study, we found significantly more deliriums in elderly patients. In a study, which recently took place in our clinic, to determine the incidence of delirium after elective abdominal surgery, an incidence of 24% was found as well as a higher mortality rate [22].

The incidence of delirium in our study was 23%.

The type of surgery also did not differ between the two groups. In both groups the same percentage of ileo- or colostomies was performed. In a study done by Lemmens et al. it was demonstrated that elderly patients with comorbidity were treated less aggressively and therefore had worse survival [23].

Remarkable in followup was that 33% of the patients that died in the elderly group died of noncolonic cancer-related causes which could be linked to preoperative comorbidities. In the younger group this was only 8%. After correction for colonic cancer-related 5-year survival, no significant difference could be found between the two groups.

## 5. Conclusion

Curative resection of colonic carcinoma in the elderly is well tolerated and age alone should not be an indication for less aggressive therapy. However, the type and number of comorbidities influence postoperative mortality and morbidity. Treatment of these comorbidities prior to surgery may influence postoperative outcome.

## Figures and Tables

**Table 1:** Patient and tumor characteristics.

	<75 year	%	>75 year	%	*P*-value
No. of patients	133	64%	74	36%	
Emergency setting	11	9%	16	22%	*P* < .05

Location of tumor					

Right hemicolon	58	44%	41	55%	NS
Left hemicolon	22	17%	6	8%	NS
Sigmoid	49	36%	25	34%	NS
Double tumor	4	3%	2	3%	NS

Invasion depth					

T1	8	6%	1	1%	NS
T2	21	16%	11	15%	NS
T3	87	65%	51	69%	NS
T4	17	13%	12	15%	NS

Dukes' classification					

A	12	9%	8	11%	NS
B	60	45%	35	47%	NS
C	61	46%	31	42%	NS

**Table 2:** Comorbidity.

Comorbidity	<75 years	%	>75 years	%	*P*-value
Other malignancy	13	10%	9	12%	NS
COPD	12	9%	10	13%	NS
Cardiovascular	33	25%	36	49%	*P* < .01
CVA	7	5%	3	4%	NS
DVT	0	0%	1	1%	NS
Hypertension	10	8%	11	14%	NS
DM	15	11%	8	11%	NS
Other*	5	4%	9	12%	*P* < .05

*rheumatoid arthritis, hyperthyroidism, hypothyroidism, and sclerodermia.

**Table 3:** Type of operation and stomata.

Type of operation	< 75 years	%	>75 years	%	*P*-value
Ileocecal resection	3	2%	3	4%	NS
Hemicolectomy right	56	42%	36	49%	NS
Transversum resection	2	1%	2	3%	NS
Hemicolectomy left	18	15%	6	8%	NS
Sigmoidresection	35	26%	16	22%	NS
Anteriorresection	13	10%	7	9%	NS
Subtotal colectomy	4	3%	1	1%	NS
Double resection*	2	1%	3	4%	NS

Stomata					

No. of primary anastomoses	115	86%	63	85%	NS
Total no. of stomata	18	14%	11	15%	NS
End ileostomy	2	10%	1	9%	NS
End colostomy	9	50%	4	36%	NS
Loop ileostomy	6	21%	3	27%	NS
Loop colostomy	1	5%	3	27%	NS

*3 patients with sigmoidresection and ileocecalresection because of danger of blow-out cecum, 2 hemicolectomy left and right because of doubletumor.

**Table 4:** Complications.

	<75 years	%	>75 years	%	*P*-value
Total no. of complications	32	24%	32	43%	*P* < .01
Anastomotic leakage	7	5%	4	5%	NS
Fascia dehiscention	5	4%	2	3%	NS
Pneumoniae	5	4%	10	14%	*P* < .05
Intraabdominal abscesses	5	4%	0	0%	NS
Delerium	21	16%	28	38%	*P* < .01
Woun dinfection	1	1%	6	8%	*P* < .05
Ileus	6	5%	6	8%	NS
Minor complications	6	5%	10	14%	*P* < .05
In-hospital mortality	6	5%	12	16%	*P* < .01

## References

[1] Greenlee RT, Hill-Harmon MB, Murray T, Thun M (2001). Cancer statistics. *Ca-A Cancer Journal for Clinicians*.

[2] Makela JT, Kiviniemi H, Laitinen S (2000). Survival after operations for colorectal cancer in patients aged 75 years or over. *European Journal of Surgery*.

[3] De Marco MF, Janssen-Heijnen MLG, van der Heijden LH, Coebergh JWW (2000). Comorbidity and colorectal cancer according to subsite and stagea population-based study. *European Journal of Cancer*.

[4] Scott NA, Jeacock J, Kingston RD (2005). Risk factors in patients presenting as an emergency with colorectal cancer. *British Journal of Surgery*.

[5] Irvin TT (1988). Prognosis of colorectal cancer in the elderly. *British Journal of Surgery*.

[6] Wobbes T (1985). Carcinoma of the colon and rectum in geriatric patients. *Age and Ageing*.

[7] Greenburg AG, Saik RP, Pridham D (1985). Influence of age on mortality of colon surgery. *American Journal of Surgery*.

[8] Violi V, Pietra N, Grattarola M (1998). Curative surgery for colorectal cancer: long-term results and life expectancy in the elderly. *Diseases of the Colon and Rectum*.

[9] (2000). Colorectal cancer collaberative group surgery for colorectal patients in elderly
patients: a systematic review. *Lancet*.

[10] Alley PG (2000). Surgery for colorectal cancer in elderly patients. *Lancet*.

[11] Faivre J, Lemmens VEPP, Quipourt V, Bouvier AM (2007). Management and survival of colorectal cancer in the elderly in population-based studies. *European Journal of Cancer*.

[12] Charlson ME, Pompei P, Ales KL, MacKenzie CR (1987). A new method of classifying prognostic comorbidity in longitudinal studies: development and validation. *Journal of Chronic Diseases*.

[13] Avital S, Kashtan H, Hadad R, Werbin N (1997). Survival of colorectal carcinoma in the elderly: a prospective study of colorectal carcinoma and a five-year follow-up. *Diseases of the Colon and Rectum*.

[14] Spivak H, Maele DV, Friedman I, Nussbaum M (1996). Colorectal surgery in octogenarians. *Journal of the American College of Surgeons*.

[15] Bouvier AM, Launoy G, Lepage C, Faivre J (2005). Trends in the management and survival of digestive tract cancers among patients aged over 80 years. *Alimentary Pharmacology and Therapeutics*.

[16] Wise WE, Padmanabhan A, Meesig DM, Arnold MW, Aguilar PS, Stewart WRC (1991). Abdominal colon and rectal operations in the elderly. *Diseases of the Colon and Rectum*.

[17] Fitzgerald SD, Longo WE, Daniel GL, Vernava AM (1993). Advanced colorectal neoplasia in the high-risk elderly patient: is surgical resection justified?. *Diseases of the Colon and Rectum*.

[18] McArdle CS, Hole DJ (2004). Emergency presentation of colorectal cancer is associated with poor 5-year survival. *British Journal of Surgery*.

[19] Setti Carraro PG, Segala M, Cesana BM, Tiberio G (2001). Obstructing colonic cancer: failure and survival patterns over a ten-year follow-up after one-stage curative surgery. *Diseases of the Colon and Rectum*.

[20] Aizawa K-I, Kanai T, Saikawa Y (2002). A novel approach to the prevention of postoperative delirium in the elderly after gastrointestinal surgery. *Surgery Today*.

[21] Van Wensen RJA, Dautzenberg PLJ, Koek HL, Olsman JG, Bosscha K (2007). Delirium after a fractured hip in more than one-third of the patients. *Nederlands Tijdschrift voor Geneeskunde*.

[22] Koebrugge B, Koek HL, van Wensen RJA, Dautzenberg PLJ, Bosscha K (2009). Delirium after abdominal surgery at a surgical ward with a high standard of delirium care: incidence, risk factors and outcomes. *Digestive Surgery*.

[23] Lemmens VEPP, Janssen-Heijnen MLG, Verheij CDGW, Houterman S, Repelaer Van Driel OJ, Coebergh JWW (2005). Co-morbidity leads to altered treatment and worse survival of elderly patients with colorectal cancer. *British Journal of Surgery*.

